# Revealing the challenges and efforts of routine immunization coverage in Nigeria

**DOI:** 10.1002/hsr2.70000

**Published:** 2024-08-20

**Authors:** Tolulope Joseph Ogunniyi

**Affiliations:** ^1^ Department of Medical Laboratory Science Kwara State University Malete Kwara Nigeria

**Keywords:** challenges, efforts, Nigeria, routine immunization coverage

## Abstract

**Introduction:**

Nigeria adopted the expanded program on immunization (EPI) in 1978, which aimed at offering children under 2 years old routine immunization (RI). Early accomplishments with the program resulted in a decrease in childhood mortality. As of 2018, Nigeria accounted for about 4.3 million out of over 13 million unvaccinated children globally. Therefore, this study revealed the challenges and efforts associated with RI program in Nigeria and the way forward.

**Methods:**

In this perspective article, I conducted searches and extracted relevant information from publicly available sources such as Google Scholar, Pubmed, and grey literature. I employed RI, challenges, efforts, and Nigeria as the keywords.

**Results:**

The 2021 Multiple Indicator Cluster Survey/National Immunization Coverage Survey reports revealed weaknesses in the program, with a national average coverage of 36%. The primary barrier to EPI across various zones is the challenge of reaching marginalized areas that were cut off from vaccination services due to operational and sociocultural issues. Some of the obstacles, such as restricted access to medical facilities, weak cold chain systems, and COVID‐19 containment strategies had a great impact on the RI program. To scale up the RI program, the Nigerian government, through the National Primary Health Care Development Agency (NPHCDA), collaborated with the World Health Organization (WHO) and Gavi, the Vaccine Alliance, to optimize the “Big Catch‐up campaign” and increase immunization coverage nationwide. By 2028, 80% of the projected zero‐dose populace is expected to be covered, reaching these eligible children with life‐saving vaccines.

**Conclusion:**

Nigeria still has a long way to go in making significant progress in the RI program. To further strengthen the immunization coverage, the country needs to maintain data on their achievements, as this will help identify gaps that need to be addressed in the immunization program.

## 1 INTRODUCTION

**Figure 1:**
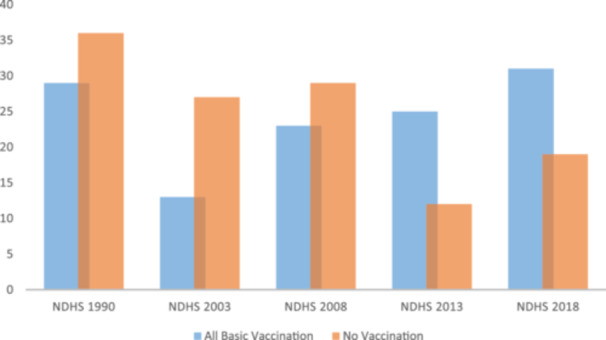
Represent the trends of immunization among age 12–23 months across the period of the Nigeria Demographic and Health Survey.^6^

Immunization is the most economical public health initiative to lower child death and morbidity.^1^ To achieve this, the World Health Organization (WHO) initiated the expanded program on immunization (EPI) in 1974 to lower morbidity and death from six vaccine‐preventable diseases.^2^ By the time a child turns 15 months old, they are expected to have received a dose of the Bacillus Calmette Guerin (BCG) vaccine, three doses of the Diphtheria, Tetanus toxoid, Hepatitis B, Pertussis, and Haemophilus influenza type B (Pentavalent) vaccine, three doses of the Oral Polio Vaccine, two doses of the Measles Containing Vaccine (MCV) and a dose of the Yellow Fever (YF) vaccine, in accordance with the WHO Childhood Revised Immunization schedule.^3^ The EPI was implemented in Nigeria in 1978, offering children under 2 years old routine immunization (RI). Early accomplishments with this program showed a decrease in childhood mortality.^1^ With an estimated 4.3 million unvaccinated children, Nigeria was the country with the highest number worldwide in 2018.^4^ In many places in Nigeria, coverage rates for regular vaccination antigens remain below 50%, even though the government offers free vaccines.^5^ According to the 2018 National Demographic Health Survey, the percentage of children who did not receive fundamental immunizations decreased from 29% to 19% between 2008 and 2018, while the rate of those who did so, grew from 23% to 31% respectively, as seen in Figure 1.^6^ Although the country has not made much progress with the RI program, this is owing to several challenges facing the program. Therefore, this study aims to reveal the challenges and efforts associated with the RI program in Nigeria and the way forward.

## 2 CHALLENGES WITH RI COVERAGE IN NIGERIA

Nigeria has struggled for many years to attain sufficient coverage of RI, a problem worsened by the coronavirus disease 2019 (COVID‐19) pandemic. The difficulties affecting RI include inefficient supply chains, subpar service provision, insufficient human capital, low demand as a result of unfavorable opinions, cultural and religious influences, disparities in finances, problems with accountability, inadequate governance, and low‐quality data.^7^ The 2021 Multiple Indicator Cluster Survey/National Immunization Coverage Survey reports reveal the weakness of the program with a national average coverage of 36%,^8^ with the following zones having the percentages of fully immunized infants: North Central 32%, North East 24%, North West 25%, South East 57%, South West 50%, and South South 49%. The primary barrier to EPI across various zones is the challenge of reaching marginalized areas that were cut off from vaccination services due to operational and sociocultural issues.^9^ The nomadic Fulani pastoralist tribes, migrant farmers, fishermen, and residents of dispersed border, and other difficult‐to‐reach villages were among the underserved populations. Ninety‐nine percent of children under five who were part of a study conducted on the Fulani nomadic tribes in the northern state of Borno had not gotten any standard immunizations or polio vaccinations.^9^

Despite making progress, Nigeria is still grappling with difficulties in establishing RI programs in places throughout its numerous states.^10^ Some of the obstacles that need to be addressed include vaccination skepticism, restricted access to medical facilities, weak cold chain systems, and insufficient financing.^10^ Another obstacle was the lack of access to healthcare services, including infrastructural issues such as poor roads and extensive travel times to medical facilities.^11^ Inadequate staffing levels due to strikes also contributed greatly to poor access and missed opportunities for vaccinating children. Additionally, adverse immunization events like pyrexia and swelling at the site of injection are of concern for children's welfare, which deters some from adhering to immunization regimens.^11^

Nevertheless, the COVID‐19 pandemic revealed weaknesses in the healthcare system, interfering with current replication initiatives and raising the possibility of undoing the gains.^7^ Increased efforts to contain the epidemic, including; enforcing lockdown and using non‐pharmaceutical therapies, resulted in a discernible decrease in vaccination rates. From July 2019 to July 2020, the state government budgetary support for providing RI and primary healthcare (PHC) services decreased from 31.8% to 24.6%.^7^ The COVID‐19 response took up more available funds, which resulted in a reduction in supportive supervision visits and RIs. The rate of BCG coverage decreased by 3.7% between July 2019 and August 2020.^7^ This revealed that before COVID‐19, significant improvements were made across all RI. Following COVID‐19 and until 2022, the country has been struggling to improve the uptake of RI as seen in Table 1.^12^

**Table 1:** National immunization coverage in Nigeria between 2018 and 2022.^12^

Vaccines	2018 (%)	2019 (%)	2020 (%)	2021 (%)	2022 (%)
Bacillus Calmette Guerin (BCG)	71	75	74	74	74
Diphtheria Tetanus Pertussis 1 (DPT 1)	69	72	70	70	70
Diphtheria Tetanus Pertussis 3 (DPT 3)	61	66	62	62	62
Poliovirus 3 (Pol3)	61	66	62	62	62
Inactivated Polio Virus 1 (IPV 1)	59	66	62	62	62
Measles Containing Vaccine 1 (MCV1)	56	58	60	60	60
Hepatitis B Vaccine (HepBB)	41	52	52	52	52
Hepatitis B Vaccine 3 (HepB3)	61	66	62	62	62
Hemophilus Influenzae type B Vaccine (Hib3)	61	66	62	62	62
Pneumococcal Conjugate Vaccine 3 (PcV3)	59	64	60	60	60
Yellow Fever Vaccine (YFV)	56	57	59	59	59

## 3 EFFORTS MADE TO STRENGTHEN THE RI COVERAGE

To address the challenges facing RI, the Nigerian government and SDG3 GAP partners worked together to establish a data system in 2020 which identified communities that were zero‐dose. This effort, spearheaded by the National Primary Health Care Development Agency (NPHCDA) and aided by UNICEF and WHO, integrated data from a range of informants, including subnational community data, health facilities, surveys, and immunization records.^13^ Following collaborative workshops and stakeholder meetings, a thorough analysis was conducted, identifying 100 local government areas (LGAs) across 18 states that are home to 1.5 million of the 2.2 million zero‐dose children in Nigeria. This investigation provided a comprehensive framework for increasing vaccination coverage and influenced the establishment of zero‐dose priority initiatives within the National Strategy for Vaccination and PHC System Strengthening. In reaching these areas, it also served as the foundation for partnership collaboration and strategies.^13^ With USD 50 million PHC and vaccination funding, GAVI collaborated directly with eight precedence states to address the high burden of zero‐dose children and insufficient immunization coverage. Through the Immunization Plus and Malaria Progress by Accelerating Coverage and Transforming Services (IMPACT) Project, the World Bank collaborated with 16 priority states, emphasizing the improvement of cold chain equipment, monitoring systems, and vaccine supplies to lower the under‐five death rate. Furthermore, 17 states were assisted in achieving targeted zero‐dose LGA by financing channels and technical support from the World Bank, UNICEF, and WHO.^13^

Over 4 million eligible children have been inoculated since March 2021, as a result of the integration of RI in COVID‐19 vaccination efforts; of them, over 700,000 received Penta‐3 vaccine as of January 2023. To create and execute a specialized and successful scheme to target zero‐dose children in environments particular to each community, NPHCDA press on with collaborating with the 100 priority zero‐dose LGAs.^13^ The Nigerian government, through NPHCDA, is working with the WHO and its collaborators, such as Gavi, the Vaccine Alliance, to optimize the “Big Catch‐up campaign” and increase immunization coverage nationwide. By 2028, 80% of the projected zero‐dose populace is expected to be covered, reaching these eligible children with life‐saving vaccines.^7^

## 4 CONCLUSION

In Nigeria, there was an early accomplishment with a decrease in childhood mortality following the implementation of EPI. Although several challenges, such as; inefficient supply chains, inadequate governance, and low‐quality data, vaccination skepticism, lack of funding, and lack of access to healthcare services, still hinder the progress of the program. The COVID‐19 pandemic further impacted the RI program owing to increased efforts to contain the epidemic resulting in a discernible decrease in vaccination rates. To further improve the RI program, the IMPACT Project, the World Bank collaborated with 16 priority states, emphasizing the improvement of cold chain equipment, monitoring systems, and vaccine supplies to lower the under‐five death rate.

## 5 RECOMMENDATION

To further strengthen the immunization coverage, the country needs to maintain data on their achievements, as this will help identify gaps that need to be fill up in the immunization program. Promoting tele‐health, community engagement and improving the communication system will further strengthen the coverage and allow achieving the target goals.

## AUTHOR CONTRIBUTIONS

**Tolulope Joseph Ogunniyi**: Conceptualization; writing—review and editing; writing—original draft.

## CONFLICT OF INTEREST STATEMENT

The author declares no conflict of interest.

## TRANSPARENCY STATEMENT

The lead author Tolulope Joseph Ogunniyi affirms that this manuscript is an honest, accurate, and transparent account of the study being reported; that no important aspects of the study have been omitted; and that any discrepancies from the study as planned (and, if relevant, registered) have been explained.

## Data Availability

Data sharing is not applicable to this article as no data sets were generated or analyzed during the current study.
